# Pelvic inclination correction system for magnetic resonance imaging analysis of pelvic organ prolapse in upright position

**DOI:** 10.1007/s00192-022-05289-0

**Published:** 2022-07-30

**Authors:** Lisan M. Morsinkhof, Martine K. Schulten, John O. L. DeLancey, Frank F. J. Simonis, Anique T. M. Grob

**Affiliations:** 1 Magnetic Detection and Imaging Group, Technical Medical Centre, University of Twente, Drienerlolaan 5, 7522 NB, Enschede, The Netherlands; 2 Multi Modality Medical Imaging Group, Technical Medical Centre, University of Twente, Enschede, The Netherlands; 3 Department of Obstetrics and Gynecology, University of Michigan, Ann Arbor, MI USA

**Keywords:** Magnetic resonance imaging, Pelvic inclination correction system, Pelvic organ prolapse, Upright

## Abstract

**Introduction and hypothesis:**

Pelvic organ prolapse quantification by means of upright magnetic resonance imaging (MRI) is a promising research field. This study determines the angle for the pelvic inclination correction system (PICS) for upright patient position, which is hypothesized to deviate from the supine PICS angle. The necessity of different PICS angles for various patient positions will also be discussed.

**Methods:**

Magnetic resonance scans of 113 women, acquired in an upright patient position, were used to determine the upright PICS angle, defined as the angle between the sacrococcygeal–inferior pubic point (SCIPP) line and the horizontal line. The difference and correlation between the upright and supine PICS angles were calculated using the paired Student’s *t*-test and the Pearson’s correlation coefficient (*r*) respectively. The effect of the difference between the upright and supine PICS angle on the measured pelvic organ extent was calculated using goniometry.

**Results:**

The mean (interquartile range) PICS angles were 29° (26–35°) for the upright and 33° (30–37°) for the supine patient position. They were significantly different (*p*<0.001) and very strongly correlated (*r* = 0.914, *p*<0.001). The 4° difference between the average upright and supine PICS angle results in an average underestimation of the measured cervix height of approximately 0.5 cm for patients scanned in upright position.

**Conclusions:**

The PICS angle for the upright patient position is 29°. The use of a dedicated PICS angle for different patient positions allows for more accurate pelvic organ extent analysis in patients with prolapse.

## Introduction

Pelvic organ prolapse is described as a downward displacement of one or more of the pelvic organs, including the bladder, uterus, bowel, and rectum [1]. As a consequence, symptoms such as vaginal bulge, pelvic pressure, and incomplete emptying or defecation may occur [1]. In clinical practice, the pelvic organ prolapse quantification (POP-Q) system is most frequently used to evaluate the extent of the pelvic organs [2]. Imaging techniques such as (transperineal) ultrasound, defecography, and magnetic resonance imaging (MRI) are used to gain additional information on, amongst others, multicompartment prolapse and functioning. MRI is the most valuable technique for quantifying the extent of prolapse because it allows concurrent visualization of multiple pelvic organs and supportive structures in relationship to the bony pelvis [3].

To perform prolapse quantification on magnetic resonance (MR) scans in a standardized way, a generally accepted quantification method is needed. Several reference lines have been defined; however, each reference line has its disadvantages, as described by Betschart et al. [4]. They therefore introduced the Pelvic Inclination Correction System (PICS) as a new reference line, based on a fixed clockwise rotation with respect to the sacrococcygeal–inferior pubic point (SCIPP) line. The PICS has the following properties:

1. Measurements of the pelvic organ position are performed along the body axis in the direction in which prolapse occurs
2. Because of the nearly horizontal direction of the PICS, distance measurements are mostly independent of the antero-posterior location of the pelvic organ
3. The pubic bone and sacrococcygeal (SC) joint, which are used as landmarks, are easily identifiable within the field of view (FOV) of the MR scan
4. The variation in orientation of the pelvis under different circumstances (e.g., rest, straining) is taken into account, which makes this reference line independent of the position of the pelvis in the MR scanner

The PICS line therefore seems to be the most valid reference line to quantify (and standardize) the extent of prolapse using MRI, and has been applied multiple times since its introduction [5–9].

In recent years, MRI acquisition with the patient in the upright position has emerged, enabling visualization of the displacement of prolapse along the body axis, which is the direction in which most prolapse symptoms occur [1]. Several studies showed that the extent of prolapse is larger when visualized in upright position than supine [10–12], even when straining is performed in supine position [13]. This indicates the importance of upright imaging to gain an accurate representation of the pelvic organ position in patients with prolapse. However, those studies used the pubococcygeal line (PCL) as the reference line, in which the variation in antero-posterior location can result in over- or underestimation of the pelvic organ height owing to the diagonal orientation of this line. Usage of the PICS line in evaluation of upright MR scans could address this issue.

The rotation angle of the PICS line is based on the average angle between the SCIPP and horizontal line of 149 women [4]. The angle was determined for the supine at rest (34°) and supine straining (29°) conditions separately. The rotation angle of the PICS line (PICS angle) in the upright position was not calculated. It is hypothesized that the PICS angle in the upright-rest condition will deviate from the supine-rest PICS angle, because of the variation in PICS angle between the supine-straining and supine-rest conditions. Therefore, the aim of this study was to determine the PICS angle for the upright-rest condition.

Because the differences between the PICS angles in the supine-rest and supine-straining condition are only 5°, one could raise the question whether it is useful to use a different PICS angle for every condition (supine-rest, supine-straining, and upright-rest), or to choose one angle and apply this to every situation. Therefore, this paper discusses the necessity of different PICS angles for different patient positions.

## Materials and methods

### Population

MR scans of women recruited for three different prolapse studies including patients and asymptomatic controls were used in this study. The studies were approved by the local ethics committees (NL57965.044.13, NL61904.091.17 and NL74061.091.20), and all women gave written informed consent in the original studies. Patients were selected from the gynecology department of the Ziekenhuis Groep Twente (ZGT) hospital in Hengelo, Medisch Spectrum Twente (MST) hospital in Enschede, and Isala hospital in Zwolle. Asymptomatic controls enrolled via flyers.

All women were 18 years or older. Women were excluded if they were unable to stand for 20 min without assistance, were not eligible to undergo an MR scan based on the MRI safety checklist, or had a jeans size ≥52 (EU) or 22 (US), because of the limited coil circumference.

Preoperative MR scans and scans without pessary were selected for analysis. If multiple scans under those conditions were available, one of the scans was selected at random. When an asymptomatic control was included in multiple studies, only the most recent MR scan was used for analysis.

### MRI examination and image analysis

All women were scanned using a tiltable 0.25T MR scanner (G-Scan; Esaote, Genoa, Italy) in supine and upright positions. The table angulation in upright position was 81° to enable patients to stand stable. In both positions a multi-slice 2D T2-weighted fast spin echo (FSE) scan was acquired in midsagittal position (echo time (TE): 2 ms, repetition time (TR): 3,480 ms, reconstructed resolution: 1.3 × 1.3 mm^2^, FOV: 340 x 340 mm^2^, matrix size: 192 x 200, slice thickness: 5 mm, number of slices: 11, total scan time: ±2 min).

**Fig. 1:**
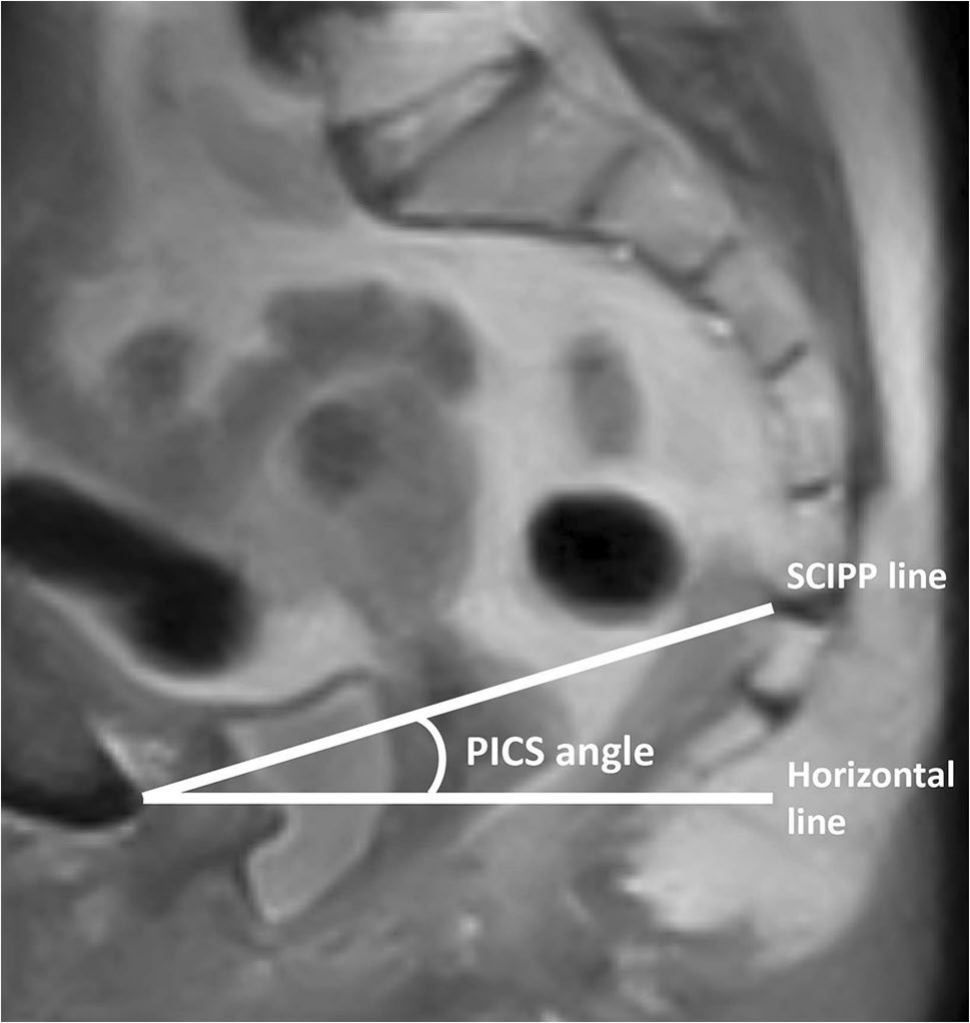
The pelvic inclination correction system (PICS) angle was defined as the angle between the sacrococcygeal–inferior pubic point (SCIPP) line and the horizontal line, based on MRI in the sagittal view. The horizontal line originates at the inferior pubic point and is oriented perpendicular to the longitudinal body axis established by the scanner bed

To determine the SCIPP line, the inferior pubic point and the SC joint (joint between the fifth sacral vertebra and the coccyx) were annotated using MATLAB 2021a (the MathWorks, Natick, MA, USA) by one researcher with 2 years experience in MR scan annotations related to prolapse (LM). If it was impossible to distinguish which vertebra was the fifth sacral vertebra because the sacral spine was partially located outside the FOV on the scan in supine position, the corresponding joint was determined by comparison with the scan in upright position. The PICS angle was determined by calculating the angle between the SCIPP line and the horizontal line, with an orientation perpendicular to the longitudinal body axis established by scanner bed and originating at the inferior pubic point, as visualized in Fig. 1.

### Statistical analysis

Statistical analysis was performed using SPSS version 28.0.1.0 (SPSS, Chicago, IL, USA). Normality of the data was assessed by visual inspection of histograms. In accordance with the methodology of Betschart et al. [4] the mean and interquartile range (IQR) of the PICS angles in supine and upright position were calculated, without differentiation between prolapse patients and asymptomatic controls. Differences between the PICS angles in upright and supine position were analyzed using the paired Student’s *t*-test. Pearson’s correlation coefficient (*r*) was used to compare the PICS angle in the supine and upright positions.

### Effect of the PICS angle difference on difference in pelvic organ distance

To evaluate the necessity of different PICS angles for different patient positions, the effect of the use of the upright instead of the supine PICS angle on the difference in distance between the organ and the PICS line (∆*d*) was calculated using the following equation:

1 $$\Delta d\equiv {d}_{upr}-{d}_{sup}= xtan\left(\Delta \alpha \right)+\frac{d_{sup}}{\mathit{\cos}\left(\Delta \alpha \right)}-{d}_{sup}$$

where *d*_*upr*_ and *d*_*sup*_ are the distance between the organ and the upright and supine PICS line respectively, *x* is the horizontal distance between the pubic bone and the pelvic organ and ∆*α* is the difference between the upright and supine PICS angle. A comprehensive overview and illustration of this equation are outlined in the Appendix.

## Results

### Demographics

**Fig. 2:**
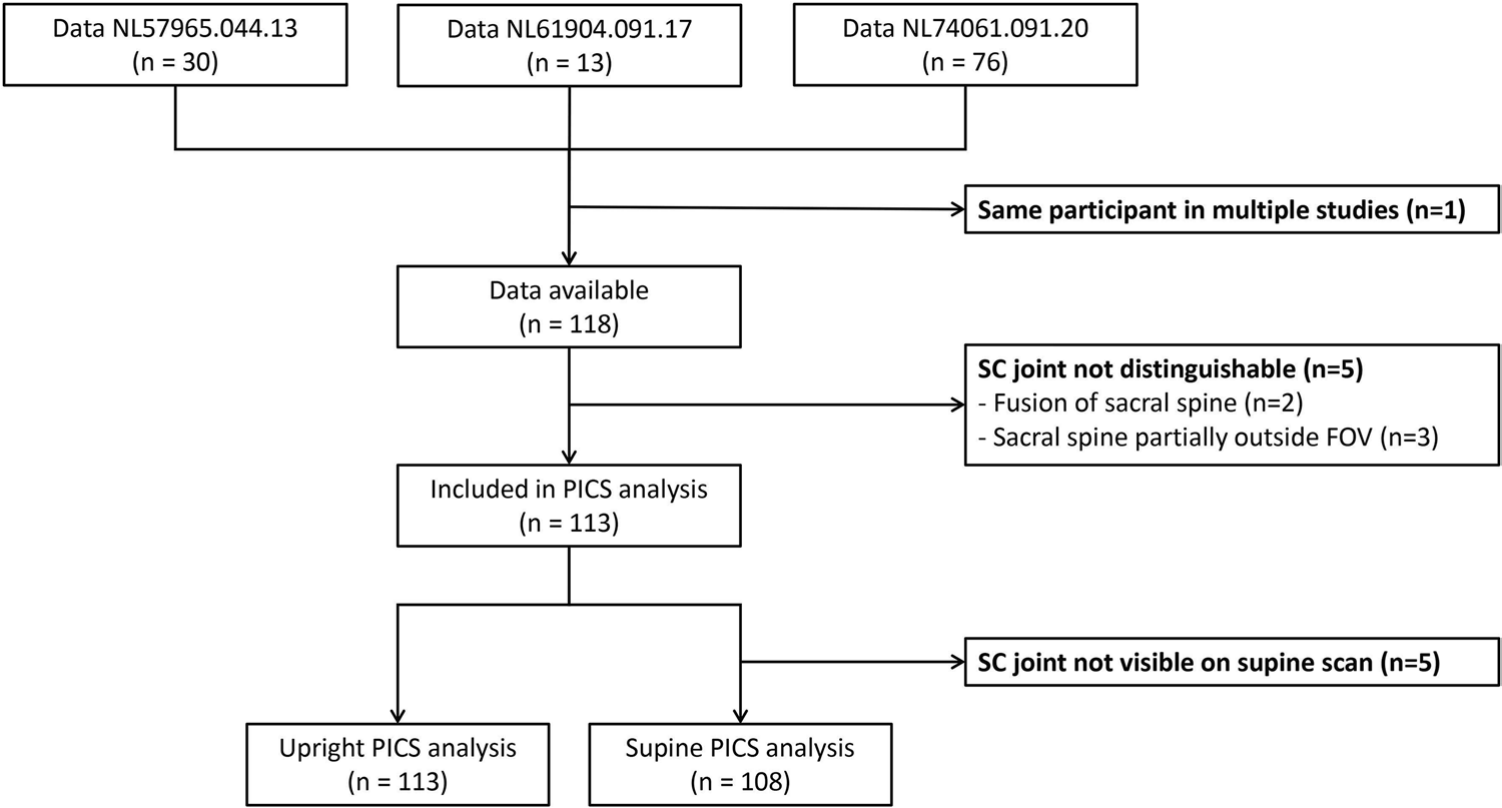
Flowchart of participants included in analysis of the upright and supine pelvic inclination correction system (PICS) angle

In total, data of 118 women were available for image analysis. Five women were excluded because it was impossible to distinguish their SC joint, because vertebrae of the sacral spine were fused (*n*=2) or partially located outside the FOV in supine as well as upright position (*n*=3). After exclusion, 113 women (56 prolapse patients and 57 asymptomatic controls) remained for analysis of the PICS angle (Fig. 2). Their median age was 57 (19–79 range) years, body mass index (BMI) 25 (18–38) kg/m^2^ and parity 2 (0–5). For analysis of the PICS angle in the supine position, an additional five scans were excluded because the SC joint was not visualized owing to a saturation band placed at the sacrum during scanning. Three women were scanned with a table angulation slightly larger than 81° (87° (*n*=1) or 90° (*n*=2)).

### PICS line

**Fig. 3:**
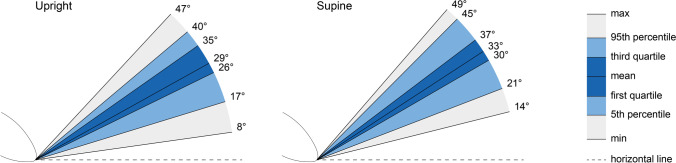
Variation in pelvic inclination correction system (PICS) angles. **a** Variation in the upright position at rest (*n* = 113). **b** Variation in the supine position at rest (*n* = 108)

**Fig. 4:**
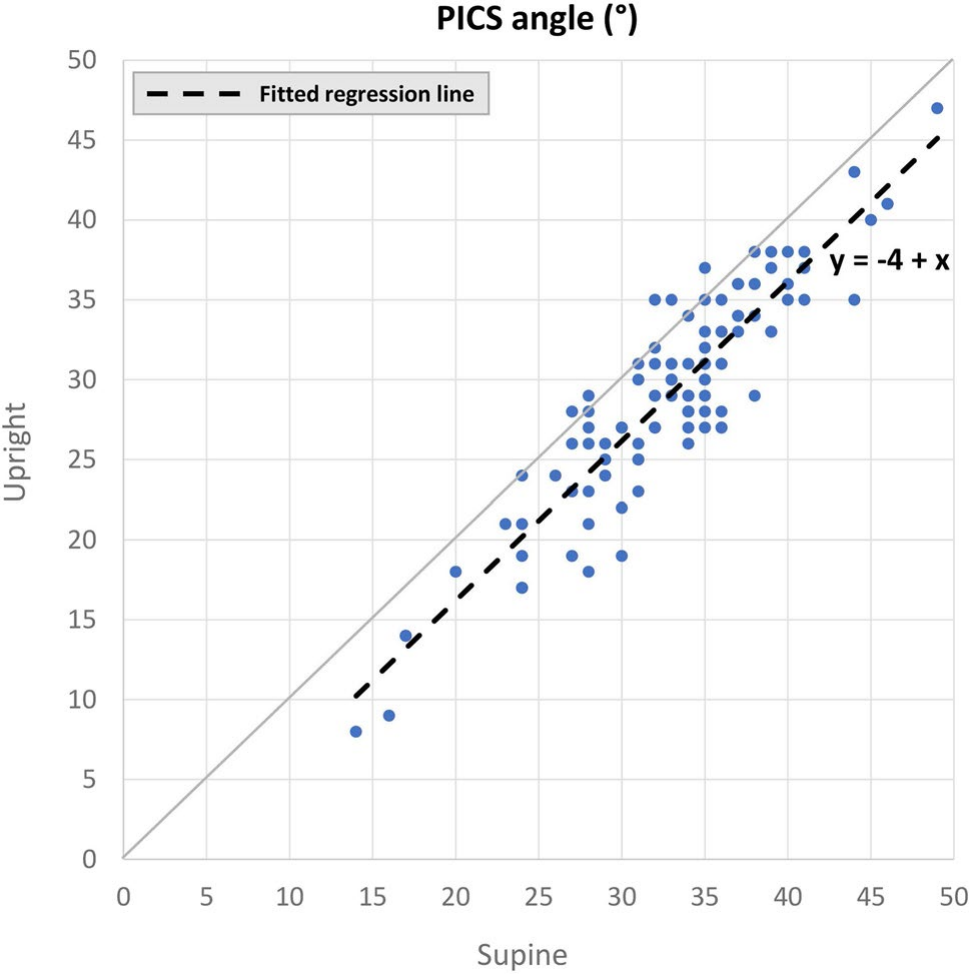
Relationship between the pelvic inclination correction system (PICS) angle in the supine and upright positions (*r* = 0.914, *p*<0.001). 89% of the data are plotted at the right of the *gray diagonal line*, indicating that in this percentage of women the upright PICS angle was smaller than the supine PICS angle. The slope of the fitted regression line of 1 indicates that if the PICS angle supine increases by 1°, the upright PICS angle also increases by 1° and vice versa

The mean calculated PICS angle for the upright position was found to be 29° (26°–35° IQR). This angle is significantly different from the angle in the supine position, which was 33° (30°–37°; *p*<0.001; Fig. 3). Pearson’s correlation coefficient indicates a very strong correlation [14] between the PICS angle in the upright and supine positions (*r* = 0.914; *p*<0.001; Fig. 4). The slope of the regression line is equal to 1, meaning that if the PICS angle supine increases by 1°, the upright PICS angle also increases by 1° and vice versa.

**Fig. 5:**
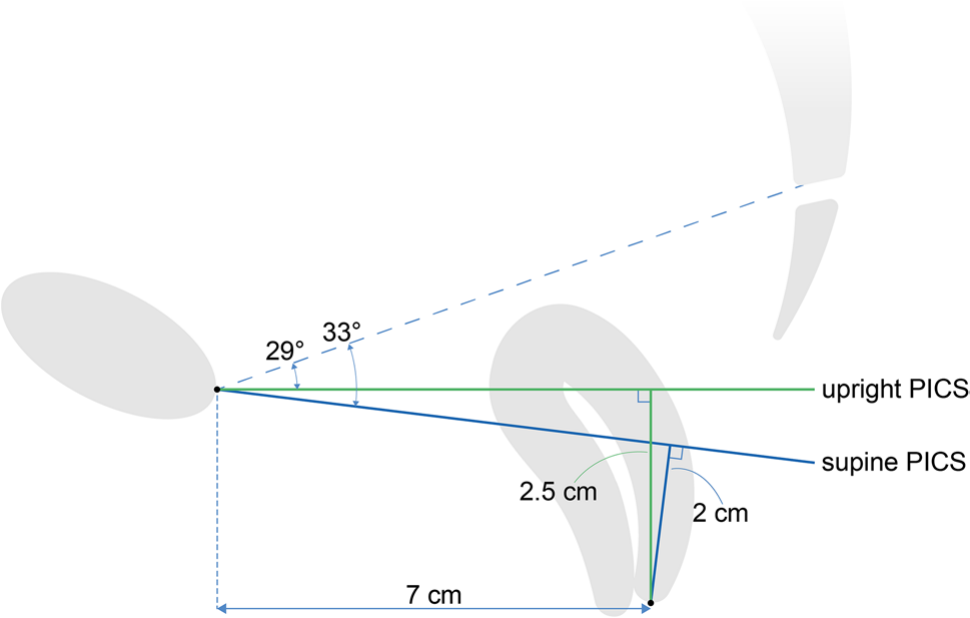
Example of the difference in measured cervix distance between the use of the upright (*green*) and supine (*blue*) pelvic inclination correction system (PICS) line, with a cervix located 7 cm posterior to the pubic bone and 2 cm caudal to the supine PICS line. There will be an underestimation of approximately 0.5 cm in cervix distance when the supine PICS line is used in the evaluation of an MR scan acquired with the patient in the upright position.

In 89% of the women the upright PICS angle was smaller than the supine PICS angle, with an average difference of 4°. According to Eq. 1, in the case of a cervix located 7 cm posterior to the pubic bone and 2 cm caudal to the supine PICS line, the difference in measured cervix height ∆*d* will be 0.5 cm. This indicates a 0.5 cm underestimation of the cervix extent when the supine PICS angle is used on measurements of prolapse extent on MR scans acquired in upright patient position (Fig. 5). Variations of *x* between 6 and 8 cm and *d*_*sup*_ between 0 and 3 cm result in variations of ∆*d* between 0.4 and 0.6 cm.

## Discussion

Prolapse analysis with the patient in the upright position, taking the descent of the pelvic organ along the body axis into account, is possible using a dedicated upright PICS angle. The current study found a PICS angle of 29° for patients in the upright position and a very strong correlation between the upright and supine PICS angles. Differences in the supine and upright PICS lines result in meaningful differences in organ location.

In reviewing the literature, no previous studies on the upright PICS angle were found. Betschart et al. [4] compared the pelvic angle, defined as the angle between the femoral heads and the sacral promontory, between MRI of women in the supine position and published measures performed in the standing position on X-ray [15]. Our study, by comparing measures in the same individuals using the same imaging modality (MRI), avoids problems of getting data from different populations.

In contrast to the upright PICS angle, we were able to compare the supine PICS angle with the current literature. Our results seem to be consistent with those of Betschart et al. [4], who found a supine-rest PICS angle with a mean value of 34° (31°–38° IQR), which is almost identical to our results. Additionally, our study results of the upright PICS angle are almost identical to those of the PICS angle at supine-straining [4]. This indicates that, in contrast to the height of the pelvic organs [13], the height of the bony structures in the upright-rest position can be compared with that in the supine-strain position.

Previous research showed a larger extent of prolapse in the upright position compared with supine [10–13]. However, the use of the supine PICS angle in the upright patient position results in an average underestimation of the measured cervix height of approximately 0.5 cm. Because in 89% of the women the upright PICS angle is smaller than the supine PICS angle, and the correlation between the upright and supine PICS angle is very strong, the use of the supine PICS angle for evaluation of MR scans acquired in the upright patient position result in a systematic underestimation of the prolapse extent in the upright position. Using the upright PICS angle instead results in a more accurate representation of the extent of prolapse in daily life. We therefore recommend using separate upright and supine PICS angles of 29° for measurements on scans acquired in the upright patient position.

The extent of prolapse is often measured by calculating the pelvic organ distance to a reference line determined in the midsagittal plane, but in practice the points of interest for the pelvic organs and the landmarks for the reference line may not be located in the same plane. To overcome this problem and allow assessment of nonmidline features such as the location of the lateral vaginal margin in estimating paravaginal descent [16], a development in the use of reference systems is the transition from two-dimensional reference lines to three-dimensional (3D) reference systems. Reiner et al. [17] introduced the 3D PICS, wherein the left and right ischial spine are used as additional landmarks to determine the orientation of a reference plane. Analysis of nonmidline features using the 3D PICS can provide insight in the effect of surgery on the different levels of pelvic organ support [18]. Moreover, the 3D PICS can be used as a reference system for pessary position and orientation [19], allowing for a better understanding of the working mechanism of pessaries. The use of the upright PICS landmarks in combination with the left and right ischial spine enables dedicated 3D analysis of the prolapse extent in the upright patient position, which can be used in multiple applications to gain more insight into prolapse treatments, such as the applications described above.

In this study we have not only introduced the upright PICS angle to enable accurate analysis of the pelvic organ extent in an upright patient position, but also calculated the supine PICS angle. This strength in the study design allows for comparison with previous literature. Some limitations must also be acknowledged. First, image analysis was performed by one researcher, thereby not considering the interobserver variability. However, as Broekhuis et al. [20] found a moderate to excellent reliability (intraclass correlation coefficient (ICC) range = 0.70–0.99) for the PCL and the landmarks of this line are comparable with the landmarks of the SCIPP line, a high reproducibility of the landmark annotation is assumed. Even though the resolution of our MR scans is lower than the resolution of the scans of Broekhuis et al. [20], because of the lower magnetic field strength, the landmarks used for calculation of the PICS angle are easily identifiable, as can be seen in Fig. 1. Second, an uncertain number (an estimated 3 out of 108) women had a cushion under their legs, which might have influenced the median PICS angle. Nonetheless, given the very small number of women involved, we assume that the effect is negligible.

Currently, information on the relationship between the outcomes of prolapse evaluation using the PICS line and the POP-Q stages used in clinical practice is lacking. Research into the relationship between the pelvic organ distances measured using the hymenal line and the POP-Q system found a high correlation [21]. Because the orientation between the PICS line and the hymenal line are comparable, it is hypothesized that the relationship between the PICS line and the POP-Q system might be similar. However, the hymen is subject to dorsal displacement during straining, thereby influencing the reference system of the POP-Q and the hymenal line, but not the PICS line. Further research should be undertaken to investigate the actual correlation between the prolapse stages determined with the upright PICS line and the POP-Q system, whereby the latter must be examined in the upright position for an accurate comparison. In this way, the advantage of the upright PICS line compared with other reference systems used during MRI analysis and clinical practice can be evaluated.

## Conclusion

This study determined a PICS angle of 29° for MR scans obtained in the upright patient position. We recommend the use of a separate PICS angle for different patient positions to enable dedicated evaluation of the extent of the pelvic organs in patients with prolapse.

## Figures and Tables

**Fig. 6:**
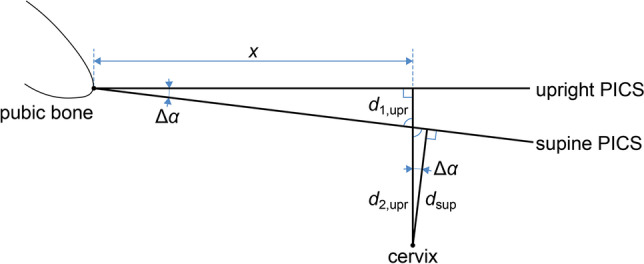
Schematic overview of the relationship between the measured pelvic organ distance using the upright (d_upr_ ≡ d_1,upr_, + d_2,upr_) and supine d_sup_ pelvic inclination correction system (PICS) angle (not to scale)

## Appendix

The difference in measured pelvic organ extent between the use of the upright and supine PICS angle is calculated based on the overview in Fig. 6. As shown in this figure, it is hypothesized that the upright PICS angle will be smaller than the supine PICS angle, as Betschart et al. [4] found a lower PICS angle during supine-straining compared with the supine-rest condition, and the supine-straining condition is used in clinical practice to imitate the upright-rest condition.

With a difference of ∆*α* between the upright and supine PICS angles, *d*_*upr*_ can be described as

$$d_{upr}\equiv d_{1,upr}+d_{2,upr}=x\tan\left(\Delta\alpha\right)+\frac{d_{sup}}{\cos\left(\Delta\alpha\right)},$$

where *x* is the horizontal distance between the pelvic organ and the pubic bone, and *d*_*upr*_ and *d*_*sup*_ are the perpendicular distances between the organ and the upright and supine PICS lines respectively. Thus, the difference in measured pelvic organ distance between the use of the upright and supine PICS angles ∆*d* will be

$$d_{upr}\equiv d_{upr}-d_{sup}=x\tan\left(\Delta\alpha\right)+\frac{d_{sup}}{\cos\left(\Delta\alpha\right)}-d_{sup}.$$
